# THE EFFECT OF CAREGIVING ON ADULT CHILDREN’S SAVING: A MULTILEVEL, CROSS-NATIONAL ANALYSIS OF 11 EUROPEAN COUNTRIES

**DOI:** 10.1093/geroni/igae098.0634

**Published:** 2024-12-31

**Authors:** Mengya Wang

**Affiliations:** Iowa State University, Ames, Iowa, United States

## Abstract

This research explores how caregiving impacts individuals’ retirement planning using Bronfenbrenner’s bioecological model. Focusing on retirement savings, it examines the association between caregiving and savings, considering gender, family support, and welfare regimes as moderators. Multi-level analysis of data from the Survey of Health and Retirement in Europe, with a sample of 3,555 individuals across 12 European countries participating in both wave one and two, reveals unexpected findings. Contrary to expectations, caregiving isn’t negatively correlated with savings, and female caregivers don’t face more financial challenges than males. Additionally, the impact of family support on savings varies by gender, with sons providing care having greater savings when they have at least one sister, while daughters engaged in caregiving tend to have fewer savings if they have at least one brother. Welfare regimes also impact savings differently based on caregiving duration; long-term caregivers in defamilialism regime (DF) experience significant savings changes between waves, while short-term caregivers in unsupported familialism regime (USF) undergo more changes. This suggests that in DF regimes, caregivers initially struggle with work and caregiving balance, but adapt successfully once arrangements are in place, possibly due to increased formal support. Conversely, in USF regimes, initial family support is strong, but over time, reliance on formal care services decreases, and family support dwindle, posing increasing long-term challenges for caregivers. This study offers insight into the implications for caregivers’ long-term financial well-being. Recognizing the financial strain they face, it’s imperative to explore effective policy options to alleviate economic burden linked to informal caregiving.

